# LoRaFarM: A LoRaWAN-Based Smart Farming Modular IoT Architecture

**DOI:** 10.3390/s20072028

**Published:** 2020-04-04

**Authors:** Gaia Codeluppi, Antonio Cilfone, Luca Davoli, Gianluigi Ferrari

**Affiliations:** Internet of Things (IoT) Lab, Department of Engineering and Architecture, University of Parma, Parco Area delle Scienze, 181/A, 43124 Parma, Italy; gaia.codeluppi@unipr.it (G.C.); antonio.cilfone@unipr.it (A.C.); luca.davoli@unipr.it (L.D.)

**Keywords:** Internet of Things, Smart Agriculture, Smart Farms, IEEE 802.11, LoRaWAN, WSN, multi-protocol gateway, heterogeneous networks

## Abstract

Presently, the adoption of Internet of Things (IoT)-related technologies in the Smart Farming domain is rapidly emerging. The ultimate goal is to collect, monitor, and effectively employ relevant data for agricultural processes, with the purpose of achieving an optimized and more environmentally sustainable agriculture. In this paper, a low-cost, modular, and Long-Range Wide-Area Network (LoRaWAN)-based IoT platform, denoted as “LoRaWAN-based Smart Farming Modular IoT Architecture” (LoRaFarM), and aimed at improving the management of generic farms in a highly customizable way, is presented. The platform, built around a core middleware, is easily extensible with *ad-hoc* low-level modules (feeding the middleware with data coming from the sensors deployed in the farm) or high-level modules (providing advanced functionalities to the farmer). The proposed platform has been evaluated in a real farm in Italy, collecting environmental data (air/soil temperature and humidity) related to the growth of farm products (namely grapes and greenhouse vegetables) over a period of three months. A web-based visualization tool for the collected data is also presented, to validate the LoRaFarM architecture.

## 1. Introduction

In recent years, a challenging trend concerning the transfer of Internet of Things (IoT)-related technologies (such as sensors, micro-controllers, network communication protocols and Cloud platforms) to Smart Farming-oriented domains, has rapidly emerged. Indeed, presently, it is not surprising to find totally automated greenhouses, in which sensor-equipped IoT nodes collect relevant environmental parameters for internal cultures and, through a data analysis stage, automatically control actuators in order to adjust air humidity [1] or soil moisture levels [2]. Similar systems have also been applied to open-field cultivation [3] and livestock management [4]. In the near future, it is thus expected that highly technological farms will be increasingly common.

The described process of technological transfer toward the agricultural sector, usually associated with terms such as Smart Farming and Smart Agriculture, is seen by several governmental organizations (e.g., The Food and Agriculture Organization, FAO [5]) as a key factor to effectively address future trends. More precisely, the achievement of an optimized and more environmentally sustainable agriculture is required to support world population growth, climate change, and water and natural resources preservation. With the perspective of supporting this transformation, the European Commission (EC) [6] has funded several projects related to Smart Farming (e.g., “Aggregate Farming in the Cloud,” AFarCloud [7]), and the adoption of IoT in the agricultural sector [8]. Moreover, the IoT represents a very promising Smart Farming enabler for several reasons. *First*, the agricultural sector has to be continuously monitored and controlled, thus leading to a huge amount of data (e.g., monitoring relevant environmental parameters for a proper plant growth) to be effectively collected, transferred, processed and stored. *Second*, in most cases the deployment of additional on-field connectivity, allowing information exchange among IoT nodes, and the use of energy scavenging (e.g., solar power) to feed them, are mandatory, since agricultural fields usually lack a wired energy supply and, often, a reliable (Internet) network coverage.

The challenges outlined in the previous paragraph can be efficiently handled through the deployment of IoT-oriented systems federated around networks of devices, collecting and forwarding (sensor) data to external processing and storage platforms (e.g., the Cloud). The achievement of this objective involves the adoption of IoT-friendly (possibly heterogeneous, in terms of transmission range and throughput) communication protocols, such as IEEE 802.15.4/802.11 and Sub-GHz technologies [9]. Regarding Sub-GHz technologies, a valuable solution is the Long-Range (LoRa) Wide-Area Network (LoRaWAN) technology [10]. In fact, since LoRaWAN is a low-power and long-range communication protocol—as will be further discussed in Section 2—it can provide connectivity over large agricultural fields, yet with low energy requirements (at the cost of a low data rate).

The purpose of this manuscript (which is an extended version of the paper “VegIoT Garden: a modular IoT Management Platform for Urban Vegetable Gardens” [3]) is two-fold: (i) to present our IoT platform for future Smart Farming scenarios, denoted as “LoRaWAN-based Smart Farming Modular IoT Architecture” (LoRaFarM), highlighting its multi-layer modularity and scalability; and (ii) to validate this architecture with a real deployment in an existing Italian farm, namely the “Podere Campáz” [11], which represents a demonstrator in the context of the H2020 AFarCloud project. The design principle of LoRaFarM aims at deploying a platform able to collect and handle relevant data for farms’ activities (e.g., growing condition of crops and greenhouse products, and livestock), further processing them for effective farm management.

LoRaFarM has a generally applicable “core” infrastructure, which can be completed with specialized *ad-hoc* modules depending on the farm’s characteristics and requirements. For example, if a farmer needs to monitor livestock habits, a *livestock module* can be introduced without a platform redefinition; the concept is true also for *crop monitoring* and *greenhouse management*. Hence, expansion modules can be added at *farm-* (or *low-*) *level*, if they include physical hardware to be installed in the deployment (i.e., sensors and actuators), as well as at *high-level*, in case data processing is needed. An example of low-level “module” consists of an IEEE 802.11-based Wireless Sensor Network (WSN), derived from the *VegIoT Garden* platform [3], to monitor the environmental parameters which influence the growth of the Podere Campáz greenhouse plants. In general, the LoRaFarM platform inherits its topological structure from the LoRaWAN architecture, since low-level communication patterns are built around the LoRaWAN technology. In detail, data coming from farm-level modules are collected by LoRaWAN-oriented End Nodes (ENs) and forwarded to a Network Server (NS) by a LoRaWAN Gateway (GW). Collected data are retrieved from the NS in order to feed high-layer modules (i.e., the Application Server, AS), and to be available (e.g., visualized) to end users.

The rest of this paper is organized as follows. Section 2 introduces a short background overview, while in Section 3 related works are discussed. In Section 4, the proposed LoRaFarM architecture is described, focusing on its modular and scalable structure. Section 5 presents an experimental performance analysis, in terms of collected data and platform energy efficiency. Finally, in Section 6 we draw some conclusions.

## 2. Background

### 2.1. Smart Agriculture

As highlighted in Section 1, modern agriculture is facing new challenges and technological transformations to cope with the emerging worldwide trends and requirements, such as urbanization and agricultural labor force aging [12]. The FAO estimates that the world population will reach 9.73 billion by 2050 and 11.2 billion by 2100 [5], with a current population of about 7.77 billion [13] (March 2020). As a logical consequence, a higher food production will be mandatory. Moreover, climatic and dietary pattern changes will intensify natural resources consumption, exacerbating critical issues such as water scarcity, land degradation, and deforestation. Other problems concern food safety, quality and traceability, since over-exploitation of natural resources and climatic change factors will lead to less nutrient food and favorable environments for food/water-borne pathogens, intensifying the use of pesticides and, consequently, potential risks for people’s health.

Therefore, leading actors in handling these trends are—now and even more in the future—digital technologies. Indeed, since the agricultural sector is characterized by unpredictability, heterogeneity and complexity, it can be better understood by monitoring, measuring and analyzing its physical parameters. This is made possible by the adoption of IoT [14].

### 2.2. Internet of Things (IoT) and Wireless Sensor Networks (WSNs)

In the context of Smart Agriculture, the IoT can be described as a large set of technologies and research disciplines oriented to support the agricultural sector, through the deployment of new data-oriented systems comprised of sensors, actuators, network connectivity, Fog- and Cloud-oriented platforms, and so on. Indeed, by collecting, forwarding, processing and analyzing relevant data coming from agricultural processes, IoT-oriented systems allow proper monitoring and management of agricultural production and farms.

From an architectural point of view, since IoT platforms are generally arranged into multiple layers, a layered organization should also be adopted in IoT/Smart Agriculture-oriented platforms. Despite several layered architectural models have been proposed to describe an IoT platform [15,16], a simple level decomposition, for IoT applications, is based on the following three layers: (i) *perception layer*, composed of devices which interact with the environment and gather data from it; (ii) *network layer*, which allows information exchange among devices and the Internet, with possible local processing; and (iii) *application layer*, which makes application functionalities accessible to the end user (i.e., the farmer). More specifically, for the agricultural domain, an IoT platform is usually built around local networks of devices (i.e., perception layer), deployed and interacting with the farm’s components, and possibly spread over a wide area, i.e., a field of wheat. This IoT platform is connected to the Internet (e.g., through Cloud-based infrastructures) or to a farmer monitoring station, where more sophisticated functionalities (such as data visualization at the application layer) can be implemented.

Local networks, belonging to the network layer and typically including nodes equipped with sensors and/or actuators, are generally organized as Wireless Sensor Networks (WSNs), which are networks composed of spatially spread sensing nodes, frequently employed to monitor and record environmental conditions. In detail, in a WSN, data are collected by a variable number of nodes (ranging from tens to hundreds or even thousands), called sensor nodes, each potentially equipped with several sensors, which measure, for example, temperature, sound, pollution levels, humidity, wind intensity, and other parameters. Gathered data generally follow a (possibly multi-hop) route to a concentrator, generally denoted as “sink” or “gateway,” which stores and/or forwards them outside the WSN. For the sake of completeness, the sink is in charge of connecting the WSN with the external world, through the use of a communication protocol which, in general, can differ from the protocol used to connect sensor nodes. In fact, information exchange between sensor nodes and the rest of the IoT platform components is often implemented integrating long-range (e.g., cellular LTE, SigFox and LoRaWAN) and low/medium-range (e.g., Bluetooth Low Energy (BLE) [17] or IEEE 802.11) communication technologies.

### 2.3. Overview of LoRa

Beside WSNs, additional relevant IoT technologies for Smart Agriculture scenarios are Low-Power Wide-Area Networks (LPWANs), being designed to offer low-cost and low-rate connectivity to a large number of constrained devices (e.g., in terms of power source and processing capabilities) geographically distributed over large areas (e.g., a farm with its surrounding fields). Two of the most recent communication technologies in the field of LPWANs are LoRa [18] and LoRaWAN [19], which are appealing choices for Smart Farming applications. In particular, the applicability of LoRaWAN is the main reason behind which LoRaFarM low-level communications are built around LoRaWAN. In detail, LoRa and LoRaWAN refer to distinct concepts: LoRa is a proprietary (and patented by Semtech Corporation, https://www.semtech.com) modulation based on Chirp Spread Spectrum (CSS) and, therefore, refers to the Physical (PHY) layer. LoRaWAN, instead, specifies the Medium Access Control (MAC) layer of an open network architecture, which is regulated by the LoRa Alliance (https://www.lora-alliance.org) and the PHY layer of which is based on the LoRa modulation.

In detail, the LoRa modulation is characterized by a Spreading Factor (SF) which defines the duration of the signal—the higher the SF, the longer the symbol time, as well as the longer the distance supported by a link. In detail, the SF may vary between 7 and 12, with SF = 12 resulting in the highest sensitivity and transmission range, but with the lowest data rate and highest energy consumption [10]—intuitively, the longer the symbol duration, the more active the radio transceiver. A decrease of one unit in SF doubles the transmission rate and halves the transmission duration, as well as the energy consumption. Since chirps at different SFs are orthogonal, the LoRaWAN Gateways (GWs) can thus receive multiple transmissions in the same frequency band with different SFs.

**Figure 1:**
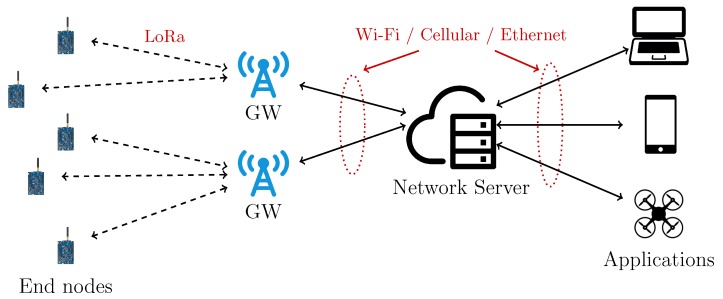
LoRaWAN architecture.

Regarding the network topology, as shown in Figure 1, LoRaWAN entails a “star-of-stars” network topology composed of ENs and GWs. The latter are in turn connected (through IP-based networks) to a Network Server (NS), which is finally connected to high-layer applications. Moreover, in LoRaWAN, the ENs can be of three types: Class A, B, and C, where the Class defines the behavior about downlink packets. In Class A, which must be supported by all LoRaWAN devices, a device can receive downlink packets only after sending a packet, thus resulting in the lowest energy consumption mode. Class B devices are suited to applications requiring a more intense downlink traffic, since they open extra receive slots at scheduled intervals by receiving a time-synchronized beacon from the GW. Finally, Class C devices are always listening to the channel: their energy consumption level is thus the highest one, but they can receive a downlink packet at any time, leading to the lowest downlink latency. GWs receive packets from all the nodes in their reception range and forward packets to the NS, which is then responsible for the management of the LoRaWAN network. A NS can handle multiple GWs—usually, it receives the same packet (originally sent by an ENs) from more than one GW.

The radio channel access in LoRaWAN is based on the ALOHA protocol: (i) an ENs wakes up and sends a packet on a selected radio channel; (ii) one or more GWs, within the transmission range of the node, receive the packet; and (iii) forward it to the NS, which eventually processes the received packets. Since in Europe LoRaWAN operates in the 868 MHz unlicensed bands, both end devices and GWs must comply with the ETSI limitation of the duty cycle (depending on the frequency, it varies from 0.1% to 10%)—unless they perform Listen-Before-Talk (LBT) or frequency-hopping techniques. Because of this constraint, each time a frame is transmitted, the *Time on Air* is calculated and, subsequently, the time in which the transmitter cannot use the channel, denoted as *time off* ($T_{\mathrm{OFF}}$), is evaluated.

To participate to LoRaWAN operations, a LoRa-compliant node must be registered and activated through the NS. LoRaWAN defines two activation methods: (i) Over-The-Air-Activation (OTAA), which is the most secure as the EN sends a *join-request* frame to the NS which, in return, (potentially) sends a *join-accept* frame; and (ii) Activation-By-Personalization (ABP), in which there is no join procedure, since the end device has all the required configuration parameters for the activation.

## 3. Related Works

The adoption of IoT technologies to support agriculture and farming is well documented in the literature and covers various fields such as to name a few, greenhouse management, crop and livestock monitoring [4,20]. As an example, in [21] the authors propose a system to collect greenhouse environmental parameters (namely temperature, light, pressure and humidity) and to visualize them thought a smartphone or a computer. In [22], IoT is combined with computer vision techniques to deploy a monitoring system for *Phalaenopsis* orchids. Otherwise, in the context of crop monitoring, in [23] an IoT system for environmental data collection and status prediction, aimed at preventing the appearance and diffusion of fungal diseases on crop fields, is proposed. Furthermore, in [24] an IoT platform, composed of open hardware and devoted to monitor mildew disease in vineyard, is presented. In the context of monitoring animals’ living environment-related variables, IoT platforms were deployed to monitor, for example, Heat Stress (HS) for cows [25], or gases, temperature, and relative humidity for bees [26].

Even if the platforms described in the previous paragraph follow a similar architectural approach (sensors deployed in the farm, data collected and forwarded to the Internet, with remote visualization/processing), they significantly differ in terms of covered scenarios, selected devices and, in particular, communication protocols. Indeed, there are solutions based on low-range technologies (e.g., BLE [25]), medium-range (e.g., IEEE 802.11 and IEEE 802.15.4 [22,23]) and long-range (e.g., cellular LTE [24,26]). The rationale behind this array of technologies lies in the requirement of choosing the most suitable connectivity type for the scenario to monitor, which depends on its size and the availability of an Internet Access Point (AP). For example, an IoT system deployed on open fields or devoted to animal localization normally needs to rely on long-range power efficient technologies (namely LPWANs), such as LoRaWAN [27], SigFox (e.g., for cow geo-localization [28]) and Narrowband IoT (NB-IoT) [29].

Most of the proposed platforms focus on the managing of a single Smart Agriculture scenario. However, since future farming is likely to cover more than one agricultural scenario, farmers may benefit by an IoT platform able to handle multiple scenarios. Furthermore, due to the fact that each scenario has its own features, the platform has to be able to integrate heterogeneous hardware and network connectivity technologies in a seamless way, in order to properly embed all of them. The descried above goals, including heterogeneity integration, abstraction and modular architecture, which are generally open challenges for the IoT [30], are addressed by the LoRaFarM platform in the context of agriculture through two state-of-the-art approaches, described in the following.

The *first* strategy involves the introduction of a *middleware* [31,32], which provides a straightforward way to access sensor data and build modular platforms, since (i) devices which produce data, (ii) collected data and (iii) high-level application are all decoupled. Moreover, it is a widely adopted approach, in fact, it is currently pursued also in the H2020 project “Aggregate Farming in the Cloud” (AFarCloud) [7]. *Second*, for what concerns the integration of IoT nodes with different communication protocols, multi-protocol gateways (namely IoT nodes equipped with multiple network interfaces) can be deployed as intermediaries between several devices and the Internet [30,33]. A similar solution, related to this topic, is proposed in [34], in which a smartphone-based multi-technology gateway, aiming at connecting Smart Objects (SOs) with different communication interfaces to the Internet, is presented.

Due to the above observations, the proposed LoRaFarM platform has been built around a core central layer (namely the middleware), which can be enriched with modules able to manage different scenarios and functionalities a generic farm may need. Furthermore, a multi-protocol gateway-based approach has been employed to manage communication protocols heterogeneity and low-level modularity. Moreover, the overall system is built around the LoRaWAN technology, due to its inherit simplicity, modularity and possibility to be deployed almost everywhere (without the need for an already existent connectivity coverage, in contrast with other long-range technologies, such as NB-IoT and SigFox), as will be discussed further in the paper. As a final remark, to the best of our knowledge, a platform with a level of integration and modularity comparable to LoRaFarM has not been presented in the Smart Farming domain, yet.

## 4. Architecture

### 4.1. The Podere Campáz Farm

LoRaFarM has been deployed and validated in a farmhouse which covers a large set of different agricultural activities, namely a biological Italian farm called “Podere Campáz” [11]. Indeed, the chosen farm covers both open-field and greenhouse cultivation together with the production of several different agricultural products. More precisely, in the “Podere Campáz” fruit, vegetables, edible flowers, herbs, and wine grapes are grown, and bees are bred. Furthermore, the farm is composed of: (i) a wide vineyard of approximately 3 ha; (ii) a greenhouse with a size of 20 × 9 × 5 $m^{3}$ (length, width, maximum height) devoted to horticultural products; (iii) some rows of tree fruit; and (iv) an area equipped with beehives. The greenhouse is manually controlled by the farmer without the support of any technology. For example, there is no system to remotely monitor the environmental parameters of the greenhouse. More precisely, the farmer manually opens and closes the greenhouse roof, in order to adjust the air humidity’s level with natural ventilation, activates irrigation sprinklers, and performs other tasks on the basis of his experience.

### 4.2. LoRaFarM Layers and LoRaWAN Architecture

As it is based on LoRaWAN, the LoRaFarM platform inherits from this network the architectural structure and the main building blocks, which are ENs, GWs, a NS, and an AS. Regarding the ENs, LoRaFarM involves devices equipped with:

- sensors (leading to sensor nodes), which collect environmental sensor data relevant for farm management (e.g., soil moisture of a field, humidity values of a greenhouse) and forward them to the Cloud (i.e., NS and applications) using the LoRa modulation;
- actuators (leading to actuator nodes), expedient to support farm automation and operations, such as field watering and greenhouse roof opening.

**Figure 2:**
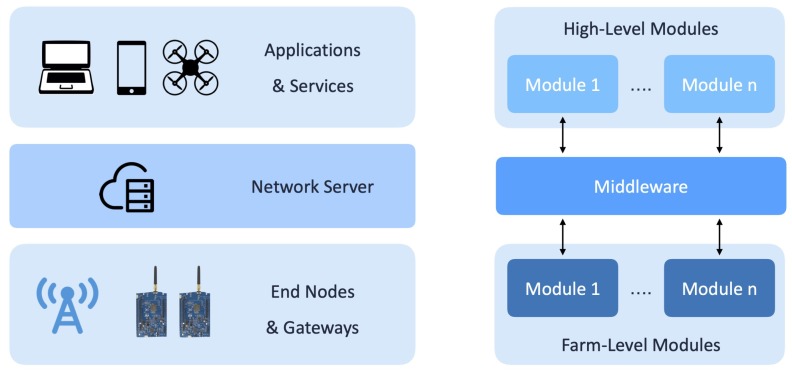
LoRaFarM platform: level decomposition and parallelism with LoRaWAN architectural components.

With respect to the functionalities and the scenario of interest, the ENs are conceptually organized in *farm-level modules*, as shown in Figure 2. In detail, these modules include all the physical devices and technologies, which are installed in the farm, applicable to expand the platform at low-level. At the moment, the platform integrates two farm modules: a *vineyard module*, useful to monitor soil parameters (i.e., soil moisture and temperature) of the farm vineyards (see Section 4.3.2), and a *greenhouse module*, which collects the environmental conditions of the greenhouse (see Section 4.3.3).

Data from and to farm modules are exchanged with the LoRaFarM platform through a LoRaWAN GW (see Section 4.3.1), which forwards messages coming from the farm (i.e., farm data) to the Internet and vice versa. Then, farm data are stored and made available to *high-level modules* thanks to the *middleware*, which connects and integrates high- and farm-level modules. With regard to the LoRaWAN architecture, the middleware layer includes the NS, which makes available farm data, and a back-end entity (namely the AS) which retrieves data from the NS and stores them into a relational database, as will be further more discussed in Section 4.4.

On the top of the middleware, data are retrieved by high-level modules, which consume them and, through proper processing operations, which depend on the functionalities implemented by the module, generate information which can be used by the farmer to optimize his/her farm management. In Figure 2, the LoRaFarM layered structure (at the right) is directly compared with LoRaWAN architectural components (at the left).

### 4.3. Farm-Level Modules

**Figure 3:**
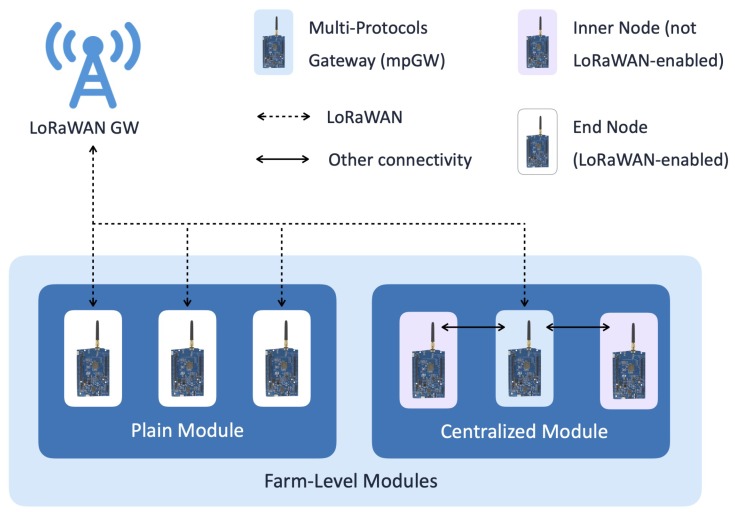
LoRaFarM architecture: the two typologies of farm-level modules.

Following the LoRaWAN paradigm, according to which a new EN can be included in a LoRaWAN network with a small number of operations (including devices registration and activation through the NS), farm-level modules can be easily added to the LoRaFarM architecture. Indeed, since a farm-level module is a network of ENs, i.e., sensor and/or actuator nodes, which are deployed in the farm and are organized according to one of the two topologies described in Figure 3, it can be smoothly integrated in the platform with a little effort. Referring to Figure 3 and to its internal network topology, a farm module can be classified as:

- *Plain Module* (PM), if it is composed of LoRaWAN-enabled nodes (i.e., ENs), receiving or forwarding data directly from or to the LoRaWAN GW;
- *Centralized Module* (CM), if it consists of some no-LoRaWAN-enabled nodes, denoted as Inner Nodes (INs), and a LoRaWAN-enabled GW (namely a mpGW).

More precisely, in LoRaFarM a mpGW is a device which supports (at least) two different communication protocols: one of them is LoRaWAN, while the other(s) may varies and is used to collect information from INs. Moreover, messages coming from and directed to INs are translated between the two (or more) protocols by the mpGW in order to enable communications between non-LoRaWAN-enabled nodes and the LoRaFarM middleware, in a seamless way. For the sake of clarity, a mpGW is connected to the platform thanks to the LoRaWAN GW, which forwards data from the mpGW (and, thus, INs) to the middleware, and vice versa.

**Figure 4:**
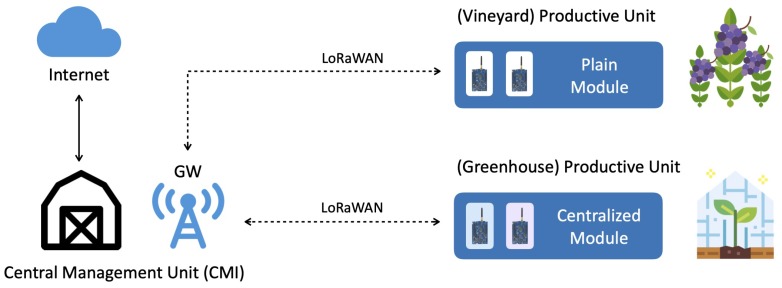
A farm organization based on “Units” (i.e., Central Management Unit, Productive Units), and the spatial distribution of the deployed farm modules and LoRaWAN GW.

The presented approach offers many advantages to LoRaFarM. *First*, heterogeneous sub-networks, in terms of capabilities (e.g., transmission range, data throughput, energy consumption), can be incorporated without altering the platform structure and, thus, making it highly scalable, flexible and suitable for a wide range of scenarios. Indeed, this gives the freedom to choose the most suitable communication protocols and traffic policy to monitor and control the farm Productive Units (PUs), such as stables, greenhouses, and fields. Moreover, the sub-networks composed of INs can be effectively managed, taking into consideration their sizes, topologies and requirements in terms of data flow. Besides its protocol translation functionality, the mpGW can be enriched with edge computing functionalities, in order to process, aggregate, and fuse sensor data. This is expedient, for example, to optimize the uplink traffic of a CM toward the LoRaWAN GW. The *second* advantage is related to the internal organization of a farm. Indeed, a farm can be seen as an aggregation of “Units,” such as a “Central Management Unit” (CMU), which may coincide with the farmer house and where the farmer remotely manages his farm through an Internet AP, and some PUs, placed far from the CMU (see Figure 4).

The PUs may either not be covered by a reliable Internet connectivity or, if covered, the available Internet access is likely to require payment for a data plan (i.e., SigFox, NB-IoT or cellular network coverage). Moreover, since the GW will likely be placed inside the CMU and connected to the available AP, the PUs will be able to exchange information through LoRaWAN technology. According to the presented approach, connectivity can be provided to a significant number of farm-level nodes, spread in the farm PUs, with the use of a single Internet AP. This last feature, together with the others outlined above, contributes to make LoRaFarM applicable, in principle, to any farm, regardless of its specific configuration.

#### 4.3.1. The LoRaWAN Gateway

Since a farm may lack a pre-existing LoRaWAN coverage, a LoRaWAN GW, based on Commercial Off-the-Shelf (COTS) devices, has been implemented and installed in the CMU of the Podere Campáz, which corresponds to the farmer house, in order to enable LoRaWAN communications. Moreover, the GW has been connected to an available Wi-Fi AP in the CMU, according to the architectural approach described in Section 4.3.

**Figure 5:**
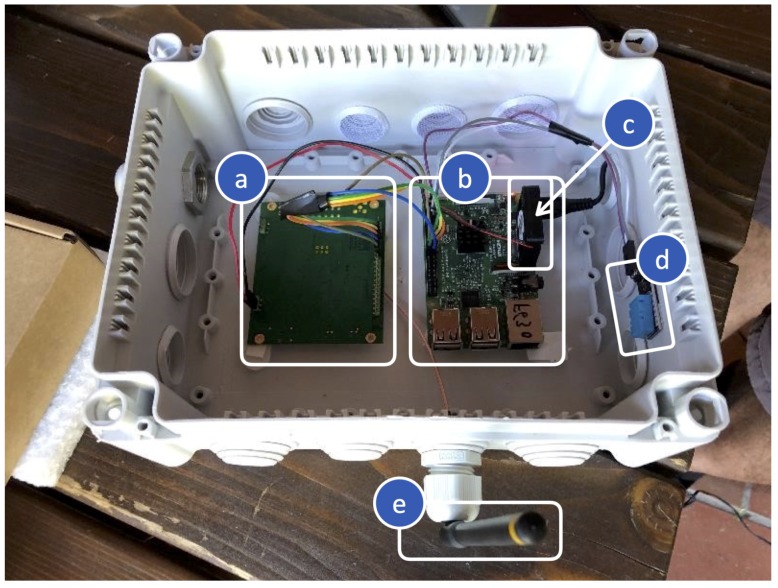
The deployed LoRaWAN GW: (**a**) iC880A-SPI concentrator board; (**b**) RPi; (**c**) the cooling fan; (**d**) the DHT11 air sensor; and (**e**) the 868 MHz Antenna.

From a technical point of view, the GW, shown in Figure 5, is based on a Raspberry Pi (RPi) [35], which an application with GW functionalities is running on. The GW is also equipped with: (i) networking components to provide the RPi with LoRaWAN-compliant connectivity (i.e., a 868 MHz Antenna and a iC880A-SPI concentrator board); and (ii) a cooling system, in order to maintain the internal temperature of the GW under control. More details on the GW hardware components and their purposes are summarized in Table 1.

#### 4.3.2. Plain Module

A PM is a farm module composed of a set of LoRaWAN-enabled devices, joining LoRaWAN network without the need for an intermediary (i.e., a mpGW) to perform operations on the collected data. For this reason, a PM is suitable to monitor and collect data from a large area which produces a small amount of information at low rate. Furthermore, beside the data gathering functionality, a PM can be designed to control actuators, according to proper decision processes remotely set at high-level. For example, a large vineyard can be covered by a PM with ENs which, equipped with sensors, measure soil moisture levels and forward sensor data to high-levels, where they are processed and employed to notify the farmer to irrigate or not the vineyard. Eventually, ENs with actuators could be deployed.

##### Vineyard Module

**Figure 6:**
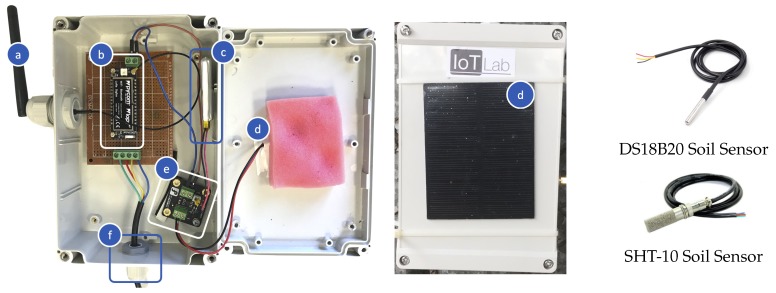
The hardware selected to implement an EN of the vineyard module: (**a**) 868 MHz Antenna; (**b**) LoPy4 board; (**c**) 3.7 V, 1800 mA LiPo Battery; (**d**) 1W Solar Panel; (**e**) Solar LiPo Charger; (**f**) Soil sensor (i.e., SHT-10 or DS18B20).

The *vineyard module* aims at collecting soil parameters, namely humidity and temperature, in order to help the farmer schedule irrigation and other operations. This module is composed of two ENs built with COTS hardware. Each EN integrates: (i) a processing unit, with a LoRaWAN transceiver and a 868 MHz antenna, which performs sensors reading, data forwarding and is based on a LoPy4 board [38]; (ii) one water-resistant soil sensor (SHT-10 or DS18B20); (iii) a power source system, composed of a rechargeable Lithium-Ion Polymer (LiPo) battery charged by a solar panel; and (iv) a protective enclosure and other materials to connect node’s hardware (as summarized in Table 2). An EN is shown in Figure 6.

The LoPy4 has been selected for several reasons: compactness, low cost, support for four connectivity types (namely LoRaWAN, SigFox, Wi-Fi and BLE), and easily programmability. Moreover, it provides natively a deep sleep mode, during which the board consumes only 25 μA. This is a relevant feature in LoRaFarM, where ENs must sleep for most of the time, waking up only for a few seconds before returning to (deep) sleep. In this way, the node battery lifetime is maximized.

The software running on the LoPy4 is written in MicroPython, which is a reduced version of Python, especially designed for micro-controllers. The main program is based on a cycle, which is periodically repeated: the node wakes up from deep sleep, reads the sensors’ values, transmits them through the LoRaWAN interface, and returns to deep sleep. The cycle time can be set to 10 min or 30 min.

The selected sensors are digital: the I2C SHT-10 sensor [39] measures both soil humidity and temperature; the One-Wire DS18B20 [40] is a soil temperature sensor. They are water-resistant, are sufficiently accurate, and support an adequate operational range of temperatures (i.e., at least, they can properly work from −40 °C to 80 °C). The power source system of a node is composed of a 3.7 V LiPo battery, with a nominal capacity of 1800 mA, which is connected to and recharged by a solar panel of 1 W through a solar LiPo charger, as shown in Figure 6.

**Figure 7:**
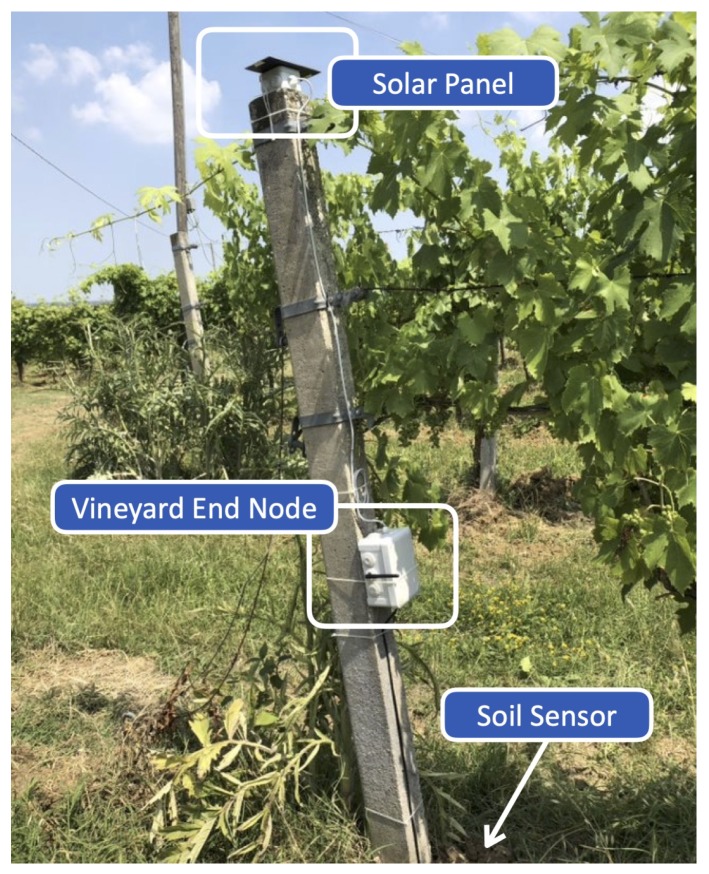
A vineyard module EN (SN-V1) installed in the farm.

In the vineyard, two nodes have been deployed: the first, denoted as SN-V1, is equipped with a SHT10 sensor; the second, denoted as SN-V2, with a DS18B20 sensor. The reason only two nodes have been installed is related to the scope of the test campaign, which is to validate the robustness of the system, in terms of board stability and suitability of selected sensors, during a limited testing period of time (i.e., three months) before starting a long-term deployment, which will include more nodes (at least six) in the following months. In Figure 7, the first installed EN (namely SN-V1) is shown.

#### 4.3.3. Centralized Module

A CM is a farm module which consists of a sub-network of INs, which are devices exchanging information with a connectivity different from LoRaWAN, in turn linked to the middleware through a mpGW. Some advantages of this approach have already been discussed in Section 4.3 and more will be discussed in the following.

First, a CM is a suitable choice for scenarios producing and/or needing to handle a large amount of data, or being able to benefit from local data processing (i.e., edge computing). Indeed, since the LoRaWAN payload packet is limited (the maximum payload length of a message is 243 Bytes) and the time interval between two consecutive message transmission is in the range of several minutes, optimizing the uplink traffic, in terms of number of sent messages and payloads’ content, is highly recommended. The optimization can be carried out by the mpGW through, for example, data fusion and aggregation. The mpGW has usually more resources than INs, e.g., it is connected to a power outlet, is equipped with a larger storage space, and has higher processing capabilities. For this reason, processing operations usually performed at Cloud level, such as controlling INs with actuators (e.g., to open a greenhouse roof, to pilot water sprinklers), can be locally delegated to the mpGW. Besides the introduced general advantages, such as relieving the workload of the platform back-end and reducing the computation latency, this approach allows the creation of “smart environments” which are self-managed even if the Internet connection of the LoRaWAN GW is absent for some hours.

##### Greenhouse Module and VegIoT Garden Integration

To monitor the greenhouse of the Podere Campáz, a CM, corresponding to an enhanced version of the Garden Wireless Sensor Network (GaWSN) of the VegIoT Garden platform [3] (whose protocol stack is reported in Table 3), has been deployed. To effectively enhance and integrate in LoRaFarM the GaWSN, some modifications, which are described and compared to the ones presented in [3] in the following, have been carried out. The GaWSN, according to the WSN nomenclature, includes three Sensor Nodes (SNs) and a Gateway Node (GN). According to the LoRaFarM notation, the GaWSN, which is still conceptually a WSN, corresponds to a CM, which is made of, respectively, two INs (instead of three SNs) and one mpGW (i.e., the GN). This notation will be adopted, in the following, to refer to the components of the GaWSN employed in LoRaFarM.

##### mpGW

The mpGW is built on a RPi equipped with a LoRa GPS HAT, which allows the RPi to join the LoRaWAN network. As a clarification, the point-to-point LoRa link between the GN and the HN of the VegIoT Garden has been replaced with a LoRaWAN link between the mpGW and the LoRaWAN GW. Furthermore, the mpGW implements an IEEE 802.11 AP to exchange information with INs and is connected to a power outlet, available in the greenhouse. The mpGW is basically a concentrator of sensor data, which are generated by INs and are aggregated before being forwarded to the LoRaWAN GW (and, therefore, to the middleware). Since the mpGW implements a reduced set of functionalities, with respect to the “powerful” board around which it is built, a RPi may seem to be under-used for the purpose, given also the reduced number of nodes installed in the greenhouse (i.e., two). However, this choice is justified by the following reasons. *First*, given that new nodes will be installed in the greenhouse during a long-term deployment, data aggregation will become more relevant. *Second*, the RPi allows easy implementation of new functionalities, which are executed locally and related to greenhouse actuation. Indeed, when the opening actuator of the greenhouse roof is installed, it will be automatically piloted by the mpGW on the basis of the collected sensor data (for example, if the air humidity reaches dangerous values, the roof is opened).

##### Inner Nodes

The greenhouse module includes two INs, which share with vineyard nodes some HW components and design principles, e.g., in terms of power source system, as detailed in Table 4. However, they there are some differences: for example, instead of a LoPy4 board, greenhouse INs are based on a Texas Instruments CC3200 Launchpad (for short, CC3200 board or CC), since they are derived from GaWSN SNs, which are based on the CC board [42].

As with the LoPy board, the CC board is also an appealing choice. Indeed, besides its built-in IEEE 802.11 connectivity and reduced dimensions, it offers three low energy consumption modes: hibernation, deep sleep, and low-power deep sleep. An experimental analysis performed with a multimeter, addressing the measurements of the energy consumption per hour of a CC during the hibernation mode (i.e., the reduced energy consumption mode considered in this work, with the lowest energy consumption), shows that its hourly energy consumption is approximately 14 mAh. In order to lower further the energy consumption of a CC board, one can remove an on-board led which cannot be switched off when the CC is powered. This allows reduction of the energy consumption from 14 mAh to 9 mAh.

**Figure 8:**
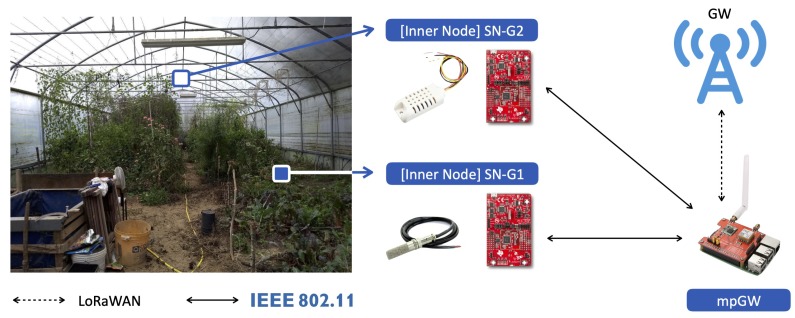
The greenhouse module: INs and mpGW with corresponding locations in the greenhouse.

Moreover, the first IN (SN-G1) is equipped with a SHT-10 sensor [39], in order to measure soil humidity and temperature, while the second (SN-G2) with an AM2032 air humidity and temperature sensor [43], as summarized in Table 2. From a software prospective, the program which runs a CC board, with respect to [3], has not been modified. The two nodes have been located in the greenhouse, according to Figure 8: (i) SN-G1 has been placed near the ground; (ii) SN-G2 has been installed at 3 m above the ground; (iii) the mpGW has been located near the greenhouse entrance and connected to an available power outlet.

### 4.4. Middleware

**Figure 9:**
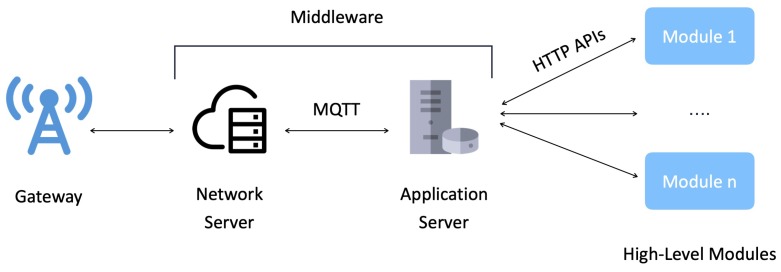
The LoRaFarM middleware layer connects farm modules with high-layer modules.

The term middleware, in the LoRaFarM domain, refers to the set of entities and technologies by which data coming from farm-level modules are collected, stored, and exposed to high-level modules. This middleware can be defined as a sort of “connecting layer” between the farm and the back-end domain and is implemented by relying on two main units: a NS and an Application Server. This implementation of the middleware is shown in Figure 9.

**Figure 10:**
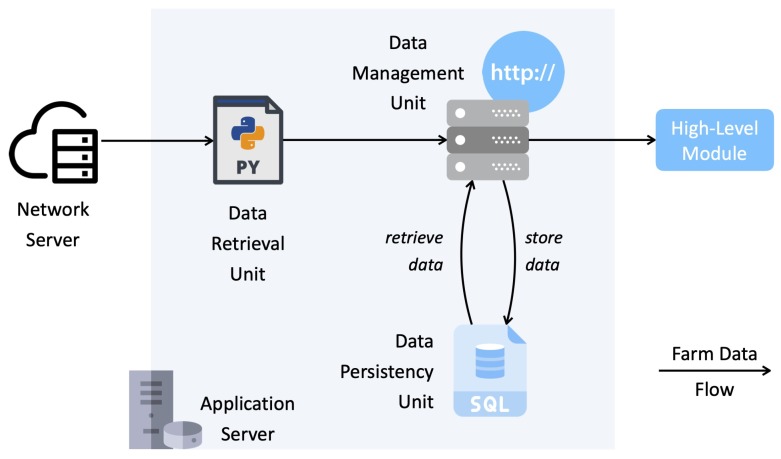
The LoRaFarM AS and the farm data flow between its components.

The NS, beside its main functionalities (in terms of ENs authentication and authorization, network encryption and decryption, and data routing), is in charge of providing the AS access to farm data, through a MQTT broker which receives data coming from LoRaWAN-enabled devices (i.e., ENs and mpGWs). The NS is built on a public network called “The Things Network” [44]. The overall structure of the AS is shown in Figure 10 and includes the following components, which implement the main functionalities related to farm data management.

- *Data Retrieval Unit*: composed by a set of MQTT clients (one per LoRaWAN-enabled device), which retrieve farm data from the NS and forward them to the Data Management Unit;
- *Data Management Unit*: stores farm data into a relational database, denoted as *Data Persistency Unit* (DPU), and makes collected data available to high-level modules through HTTP APIs.

With regard to the DPU, the choice behind the adoption of a relational model-oriented database as storage paradigm has been justified by the need to create a well-structured and general-purpose first-level repository, which could record collected information being the basis to build higher-layer applications. Indeed, the DPU also stores LoRaWAN packets forwarded by the deployed nodes and these data may be helpful, for example, to conduct diagnostic and performance analyses on the platform LoRaWAN network.

Nevertheless, the storage technology should be selected taking into account the amount of data to be persistently maintained and their correlation. Although the deployed SQL-based database properly fits the current requirements of LoRaFarM (in terms of amount of data), the LoRaFarM platform could be extended to use other storage technologies. For instance, non-relational (NoSQL)-oriented storage paradigms, as well as time series database, are possible choices. This extension should allow to better organize and distribute data storage capabilities among multiple and more specialized systems (i.e., among SQL and time series databases, as well as distributing data on database farms far from each other). Please note that since time series are probably the simplest storage approach for sensor data, high-layer modules requiring access to sensor data may benefit from this storage paradigm.

### 4.5. High-Level Modules

High-level modules are responsible to interface the platform with the farmer, in a modular and customizable way. Since, as mentioned before, farm data can be easily retrieved with HTTP APIs from the middleware, *ad-hoc* modules can be independently developed in order to satisfy the particular needs of a target farm, without altering the existing platform infrastructure but simply adding them on top of the platform. Two illustrative high-level modules, which have already been integrated into the platform, corresponding to a website and the VegIoT Garden mobile App, are presented in Section 4.5.1 and Section 4.5.2, respectively.

#### 4.5.1. Data Visualization Dashboard

**Figure 11:**
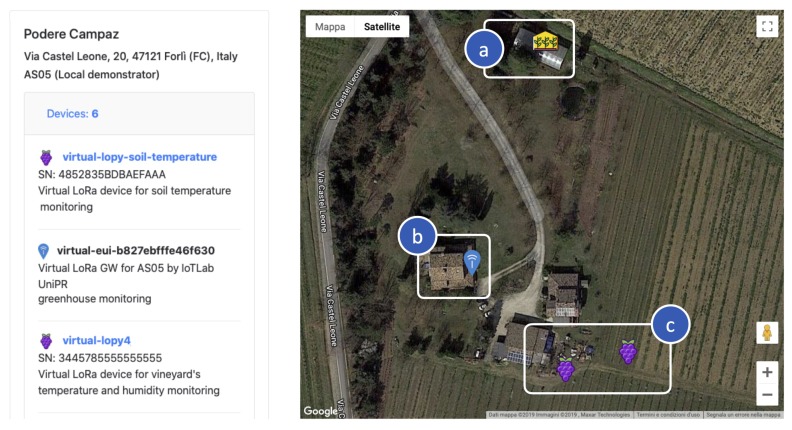
The website homepage displaying the deployed nodes and their location in the farm: (**a**) two INs and a mpGW in the greenhouse; (**b**) the LoRaWAN Gateway; and (**c**) two ENs in the vineyard.

**Figure 12:**
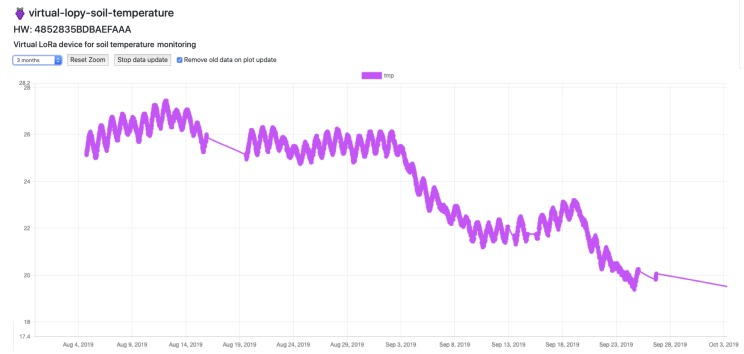
The developed dashboard: soil temperature values collected during 3 months of test by the SN-V2 of the vineyard module.

To validate LoRaFarM as a monitoring platform and to visualize collected data, a web-based dashboard has been implemented. Moreover, as shown in Figure 11, in the dashboard the deployed nodes and their locations in the farm are shown over a map. In Figure 12, soil temperature values, obtained from a 3 month data harvesting campaign with the SN-V2 EN of the vineyard module, are shown through a plot chart.

#### 4.5.2. VegIoT Mobile App Integration

Beside the web-based dashboard, the platform can also be enriched with the introduction of the VegIoT Garden mobile App [3]. This App has several functionalities: (i) visualization of farm data with a mobile device (e.g., a smartphone); and (ii) definition of alarms and settings allowing the farmer to be notified if sensor data reach values beyond properly set thresholds. The integration of the VegIoT App inside the LoRaFarM platform requires only the deployment of a Constrained Application Protocol (CoAP) Server module, in order to make the collected data, which are retrieved from the middleware, available to the mobile app.

Finally, it can be remarked that the simplicity and the small number of operations required to add a high-level module, as the VegIoT app, into the platform are a consequence of the platform architecture and, more precisely, are due to the decoupling, from physical hardware and back-end functionalities, performed by the middleware.

## 5. Experimental Results and Discussion

To experimentally validate the platform, in terms of data collected and energy consumed by platform nodes, a 3 month validation campaign was performed. As described in Section 4, two farm modules, each connected to two sensor nodes (namely two INs and two ENs, for vineyard and greenhouse modules, respectively) gathering sensor data related to environmental parameters to be monitored, were installed in the Podere Campáz. Sensor data were collected with a sampling interval equal to 10 min or 30 min and stored, at middleware level, into a MySQL database for further processing. The collected data are presented and discussed in Section 5.1. In Section 5.2, the lifetime of the used sensor nodes is evaluated both theoretically and experimentally. This is very important as sensor nodes need to be installed in the farm and be operational for several months (or years) without the need to replace batteries.

### 5.1. Collected Data

As mentioned above, two INs have been installed in the greenhouse to monitor soil and air humidity and temperature (i.e., SN-G1 and SN-G2, respectively) and two ENs have been deployed in the vineyard to measure soil temperature and humidity (namely SN-V1) or soil temperature (namely SN-V2). The collected data will be presented and discussed in Section 5.1.1 and Section 5.1.2, respectively.

As introduced before, nodes collect data with a sampling interval equal to 10 min, with the exception of SN-V1, which transmits data every 30 min. This mismatch has been introduced for the following two reasons. First, it is interesting to verify if a long sampling interval is sufficient to properly monitor environmental parameters, as this will allow the support of a larger number of deployed sensor nodes, keeping the network traffic, which depends on the number of messages sent by a node every hour, manageable. Second, it is interesting to evaluate if the throughput (i.e., the percentage of successful transmissions) depends on the sampling interval. For this reason, the numbers of successful and lost packet data transmissions from the deployed nodes, with the corresponding success rate, the distance from the GW and the employed SF (which has been fixed to SF7), are summarized in Table 5.

As shown in Table 5, the success rate is higher than 80% for almost every node, with the exception of SN-G1, for which it is about 70%. A possible reason behind this result is due to the operative conditions and the location where the SN-G1 has been deployed. Indeed, since it has been installed in the greenhouse close to plants’ leaves, the sun light is received by the node’s solar panel with sub-optimal conditions: this influences recharging of the node’s battery and the insufficient energy has a negative impact on the transmission. Furthermore, even if SN-G2 has been deployed in the same scenario of SN-G1, its position (namely near the greenhouse roof, without surrounding vegetation) allows better recharging and this leads to higher success rate, which is approximately equal to 83%.

A last consideration concerns the similarity of the success rates of vineyard nodes, which transmit with different sampling intervals—more exactly, 10 min for SN-V1 and 30 min for SN-V2. As shown in Table 5, their success rates differ only by around 1%.

#### 5.1.1. Greenhouse Collected Data

**Figure 13:**
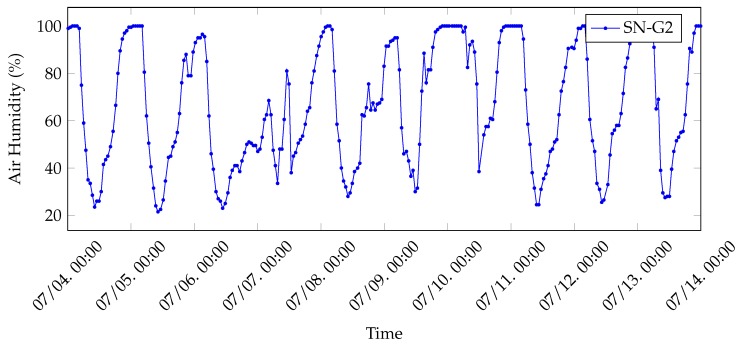
Air humidity related which has been collected in the greenhouse during ten days of test (by the SN-G2 node).

The air humidity data collected by SN-G1 during 10 days of test are shown in Figure 13. As expected, air humidity tends to 100% during night hours (from 11 pm to 4 am): this corresponds to the time interval during which the greenhouse is closed and, thus, no external air comes in. Otherwise, it decreases from approximately 4 am to 10 am and then rises again, following a periodic cycle on a daily basis. As a remark, the greenhouse is opened manually by the farmer every day at about 4 am, thus motivating the sharp decrease of air humidity around that hour.

#### 5.1.2. Vineyard Collected Data

**Figure 14:**
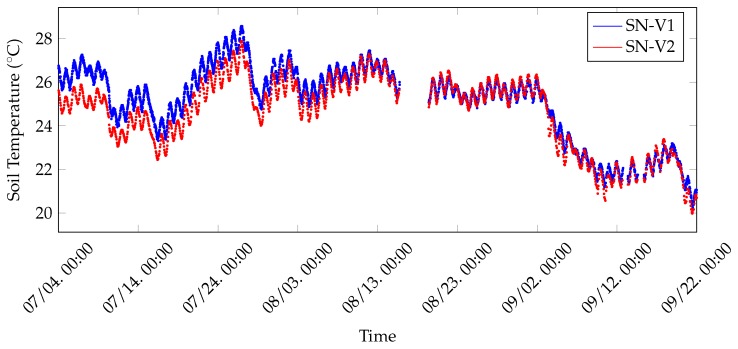
Soil temperatures coming from vineyard nodes (i.e., SN-V1 and SN-V2), which have been collected during three months of test in 2019.

With reference to Figure 14, soil temperatures related to vineyard and, thus, coming from SN-V1 and SN-V2, have been collected for approximately 3 months. As can be inferred from Figure 14, data coming from these two nodes follow a similar periodic trend on a daily basis, in which soil temperature reaches higher values during night hours and lower during light hours. The consideration remains valid during all months of testing (namely July, August and September 2019). Although in the first half of the chart data collected by the two nodes seem to be vertically shifted by approximately 1 °C, in the other half the difference between them decreases.

**Figure 15:**
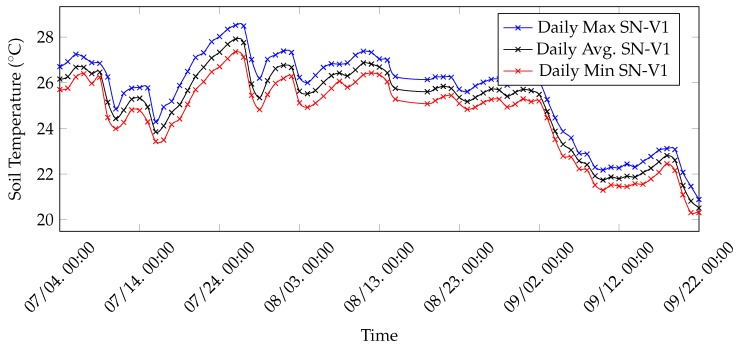
Minimum, maximum and average daily soil temperatures collected by SN-V1 during three months in 2019.

**Figure 16:**
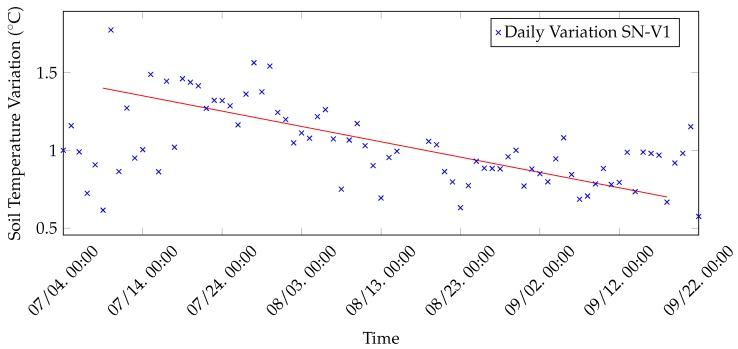
Daily soil temperature variation (namely differences between daily maximum and minimum temperature) of SN-V1 measurements, during three months of test in 2019.

Moreover, collected data have been employed to discover the daily minimum, maximum and average soil temperature for vineyard nodes. Since soil temperatures seem to follow the same trend, then daily minimum, maximum and average values have been calculated exclusively for SN-V1 data and are shown in Figure 15. Node SN-V1 has been selected, rather than SN-V2, because SN-V1 transmits every 10 min and, thus, the results are richer. As can be expected, minimum, maximum and average temperatures tend to decrease from July to September. The observation is applicable also to the daily temperature variation, which has been computed, for each day, as the difference between the registered maximum and minimum temperatures. Indeed, as shown in Figure 16, the daily temperature variation tends to drop from July to September.

### 5.2. Energy and Power Consumption

Two main aspects are relevant, from an energy consumption point of view, in the deployment of an IoT platform: (i) the selection of a proper type of energy source to feed network nodes; and (ii) the optimization of the energy use during node’s lifetime. Indeed, since in several IoT scenarios, such as Smart Agriculture, the employment of power outlets is generally unfeasible, appealing approaches involve the use of batteries and/or power scavenging techniques (e.g., sun light, electromagnetic waves, etc.). Moreover, the energy provided by “constrained” energy sources, in terms of output power, has to be carefully managed, in order to avoid, for example, the use of batteries that need to be often replaced.

The considerations in the previous paragraph apply to the LoRaFarM platform: since its sensor nodes have to be deployed outdoor and need to be operational for months without maintenance, they are powered by LiPo batteries recharged by a solar panel, as described in Section 4. Furthermore, in order to verify the optimality of the chosen power source, energy autonomy and average current consumption of the battery-operated nodes of LoRaFarM (namely INs and ENs) are investigated. More in detail, a theoretical estimation of a node lifetime, evaluated while it is powered only by a LiPo battery, is discussed in Section 5.2.1, whereas experimental results, related to the combination of the LiPo battery and the solar panel, are presented in Section 5.2.2. As a definition, the energy autonomy can be seen as the operational autonomy of a sensor node, based on its LiPo battery capacity and operational pattern. From a time perspective, the energy autonomy corresponds to the node’s lifetime.

#### 5.2.1. Theoretical Energy Autonomy Analysis

To have a clear idea concerning the energy requirements of a LoRaFarM sensor node, its lifetime, corresponding, to the number of working days/hours until the battery runs out of charge, when powered only by a LiPo battery, has been calculated, for both greenhouse INs and vineyard ENs, thus following the approach presented in [3].

First, experimental values, related to the INs and ENs’ average current consumed and time duration of their operational states (activity and inactivity/sleep), have been measured and are summarized in Table 6.

Second, the average consumed currents by, respectively, INs and ENs, can be calculated as follows:

(1) $$I_{IN_{\mathrm{mean}}}=\frac{t_{IN_{\mathrm{work}}}\;\cdot \;I_{IN_{\mathrm{work}}}+t_{IN_{\mathrm{sleep}}}\;\cdot \;I_{IN_{\mathrm{sleep}}}}{t_{IN_{\mathrm{work}}}+t_{IN_{\mathrm{sleep}}}}\approx 9.57\mathrm{mA}.$$

(2) $$I_{EN_{\mathrm{mean}}}=\frac{t_{EN_{\mathrm{work}}}\;\cdot \;I_{EN_{\mathrm{work}}}+t_{EN_{\mathrm{sleep}}}\;\cdot \;I_{EN_{\mathrm{sleep}}}}{t_{EN_{\mathrm{work}}}+t_{EN_{\mathrm{sleep}}}}\approx 0.891\mathrm{mA}.$$

Under the assumption that a sensor node is powered by a 3.7 V LiPo battery with a capacity $C_{\mathrm{LiPo}}$, the node’s expected lifetime (equivalently, its energy autonomy) can be expressed as:

(3) $$t_{EN/IN}=\frac{C_{\mathrm{LiPo}}}{I_{EN/IN_{\mathrm{mean}}}}.$$

As an example, with a battery with a capacity $C_{\mathrm{LiPo}}$ = 1800 mAh, the theoretical expected lifetimes, calculated according to Equation (3) for an IN and an EN, are approximately 8 days and 84 days, respectively.

Our results show that the estimated lifetimes vary significantly between INs and ENs: more precisely, the lifetime of an EN is approximately an order of magnitude higher than that of an IN. The reason behind this difference is due to the characteristics of the boards used in the nodes (i.e., LoPy4 for ENs and CC for INs). More specifically, the difference is due to the consumed current, mainly during their inactivity state (see Table 6), which is 0.05 mA and 9 mA, for LoPy4 and CC, respectively. Indeed, since nodes sleep for at least 98.5% of the time, the power consumed during their inactivity state is the main feature which influences a node’s lifetime.

To conclude, from a practical point of view, the nominal capacity of a battery cannot be completely employed by a node, since its potential tends to decrease with use. This is due to the fact that for all boards and devices, there exists a voltage value (namely 3.5 V for the LoPy4 [38] and 2.5 V for the CC [45]) below which they stop working correctly: this shortens the actual lifetime of platform nodes, as has been experimentally demonstrated in [3].

#### 5.2.2. Practical Energy Autonomy Considerations

The results shown in Section 5.2.1, in terms of node’s lifetime, justify the adoption of a solar panel to recharge a node’s battery in order to extend its lifetime in the range of months (and years). Indeed, under certain environmental conditions, such as bright days with average temperatures of about 25 °C, a solar panel of just 1 W, correctly oriented towards the sun, can completely recharge a 3.7 V LiPo battery of 1800 mA in about 13–14 h. For this reason, a sunny summer day is usually sufficient to fully recharge the chosen LiPo battery. Moreover, even if nodes have to work in worse conditions, e.g., cloudy days, sub-optimal solar panel orientation, or installation in closed environment as greenhouses, the same recharging performance is typically achieved in two or three days.

As a final remark, given the amount of energy hourly consumed by a working platform node (which is approximately 9/0.9 mAh, for INs/ENs, respectively), it can correctly operate for multiple months. In fact, even if its battery completely discharges (e.g., there are several consecutive days with bad weather), once it regains a sufficient battery charge (e.g., owing to one or more sunny days), a LoRaFarM node can restart transmitting sensor data without external intervention. These considerations have been practically validated since the platform nodes have correctly forwarded data for 81 days, with no additional action, even in the presence of sub-optimal weather conditions for the battery recharging.

## 6. Conclusions

We have proposed an IoT-oriented platform, denoted as LoRaFarM, aimed at supporting the management of an arbitrary farm through the integration of heterogeneous IoT technologies, such as communication protocols and COTS HWs, enabling the collection, exchange, processing, and visualization of relevant farm data. Moreover, being LoRaFarM (i) based on the LoRaWAN architecture, (ii) built around a core middleware layer, and (iii) enriched with *ad-hoc* modules (independently developed, customized and integrated into the platform) at high or low level (in order to manage specific farm scenarios), it can find a wide applicability in the Smart Farming domain.

To experimentally evaluate the proposed platform, the LoRaFarM architecture has been deployed in an Italian farm (Podere Campáz) and evaluated, in terms of collected data and nodes’ energy efficiency, over a 3 month time period. More precisely, four sensor nodes (ENs and INs) have been installed in the greenhouse and in the farm vineyard in order to collect relevant environmental parameters for vegetables and grape plants growth (i.e., air and soil humidity and temperature). Moreover, in order to let the farmer able to visualize the sensed gathered data, a web-based dashboard has been developed. Finally, from a practical point of view, the deployed nodes, which are fed by a solar panel-recharged LiPo battery, have correctly transmitted data for 3 months, both in outdoor (i.e., vineyard field) and indoor (i.e., greenhouse) environments and despite bad weather conditions. Indeed, even though some samples have been lost during a couple of rainy days, once their batteries have been recharged by solar light during the following sunny days, nodes restarted to transmit properly.

An interesting future research direction may be related to enhanced data analysis predicting the evolution of environmental parameters to prevent plant diseases, relying on Artificial Intelligence (AI) and Machine Learning (ML) techniques. Other activities may involve the introduction of a microservices architecture at middleware layer, as well as the definition of additional low-level modules built around other communication protocols (e.g., BLE) and able to monitor other environmental parameters, such as air quality, in order to extend LoRaFarM with new functionalities. 

## Figures and Tables

**Table 1:** The deployed LoRaWAN GW: selected hardware and components’ functionalities.

Block Name	Component Name	Component Details
Processing Unit	RPi	Low-cost single-board computer [35]; core of the GW where the programs implementing the GW functionalities are executed.
Networking Unit	868 MHz antenna	Allows transmission and reception of the data in the 868 MHz band.
	Pigtail for antenna	Connects the antenna with the iC880A-SPI concentrator board.
	iC880A-SPI	Concentrator board [36] which enables parallel LoRaWAN communications over multiple channels and with different SFs; it is connected to the 868 MHz Antenna and the RPi; allows data to be transmitted and received with LoRa by the RPi.
Cooling Unit	DHT11	Low-cost, small, digital, air temperature and humidity sensor [37]: used to monitor the internal temperature of the GW and, eventually, to turn it off in case of too high temperatures.
	Cooling fan	Small fan cooling connected to the RPi and activated to lower the internal temperature of the GW, if necessary.
Power source system	RPi wall cube power adapter	Connects the RPi to a power outlet.
Other components	IP66 box; cables; jumpers.	

**Table 2:** Vineyard module ENs: building blocks and selected hardware.

Block Name	Component Name	Component Details
Processing Unit	LoPy4 board	Functionalities: duty-cycling (period of 10 min or 30 min) between short activity (sensor reading and data transmission) and deep sleep [38].
	868 MHz antenna	Enables data transmission over LoRaWAN in the European free license bandwidth; connected to the LoPy4 board.
One soil sensor	SHT-10	Digital sensor; water resistant; I2C communication protocol; measures soil humidity and temperature. [39]
	DS18B20	Digital sensor; water resistant; One-Wire communication protocol; measures soil temperature. [40]
Power source system	LiPo battery	Voltage: 3.7 V; capacity: 1800 mA.
	Solar LiPo charger	Allows LiPo battery to be recharged by a solar panel. [41]
	Solar Panel	Power: 1 W; size: 80 × 100 mm.
Other components	Protective enclosure	Includes one IP66 box and two cable claps.
	Cables; jumpers; veroboards; female pin headers; terminal blocks; mammut terminals.	

**Table 3:** IoT-oriented protocol stack adopted inside the greenhouse module (right) with corresponding reference ISO/OSI layers (left).

Application	CoAP
Transport	UDP
Network	IP
Datalink	IEEE 802.11
Physical	IEEE 802.11

**Table 4:** Greenhouse module INs: building blocks and selected hardware.

Block Name	Component Name	Component Details
Processing Unit	CC3200 Board	Functionalities: duty-cycling (period of 10 min) between short activity (sensor reading and data transmission) and deep sleep [42].
One soil sensor	AM2032	Digital sensor; One-Wire communication protocol; measures air humidity and temperature [43].
	SHT-10	Digital sensor; water resistant; I2C communication protocol; measures soil humidity and temperature [39].
Power source system	LiPo battery	Voltage: 3.7 V; capacity: 1800 mA
	Solar LiPo charger	Allows LiPo battery to be recharged by a solar panel [41].
	Solar Panel	Power: 1 W; size: 80 × 100 mm.
Other components	Protective enclosure	One IP66 box and two cable claps.
	Cables; jumpers; veroboards; female pin headers; terminal blocks; mammut terminals.	

**Table 5:** Nodes deployed performance in terms of successful and lost observations during 81 days of test.

Node Name	Location	Successful	Lost	Total	Success Rate	Distance from GW (m)	SF
SN-G1	greenhouse (soil)	8167	3497	11,664	70.02%	93	SF7
SN-G2	greenhouse (air)	9677	1987	11,664	82.96%	90	SF7
SN-V1	vineyard (soil)	9486	2178	11,664	81.33%	54	SF7
SN-V2	vineyard (soil)	3197	691	3888	82.23%	125	SF7

**Table 6:** Experimental results on mean current consumption and time duration of platform nodes during their operational phases (activity and inactivity/sleep).

State	Average Current (mA)		Duration (s)	
	End Nodes	Inner Nodes	End Nodes	Inner Nodes
Activity	$I_{EN_{\mathrm{work}}}$ = 73	$I_{IN_{\mathrm{work}}}$ = 47.2	$t_{EN_{\mathrm{work}}}$ = 7	$t_{IN_{\mathrm{work}}}$ = 9
Inactivity/sleep	$I_{EN_{\mathrm{sleep}}}$ = 0.05	$I_{IN_{\mathrm{sleep}}}$ = 9	$t_{EN_{\mathrm{sleep}}}$ = 600	$t_{IN_{\mathrm{sleep}}}$ = 600

## Abbreviations

ABP	Activation-By-Personalization	IoT	Internet of Things
AFarCloud	Aggregate Farming in the Cloud	LiPo	Lithium-Ion Polymer
AI	Artificial Intelligence	LoRa	Long Range
AP	Access Point	LoRaFarM	LoRaWAN-based Smart Farming Modular IoT Architecture
AS	Application Server	LoRaWAN	LoRa Wide-Area Network
BLE	Bluetooth Low Energy	LPWAN	Low-Power Wide-Area Network
CC	TI CC3200 Launchpad	MAC	Medium Access Control
CM	Centralized Module	ML	Machine Learning
CMU	Central Management Unit	mpGW	multi-protocol GW
CoAP	Constrained Application Protocol	NB-IoT	Narrowband IoT
COTS	Commercial Off-The-Shelf	NS	Network Server
CSS	Chirp Spread Spectrum	OTAA	Over-The-Air-Activation
DPU	Data Persistency Unit	PM	Plain Module
EN	End Node	PU	Productive Unit
GaWSN	Garden Wireless Sensor Network	RPi	Raspberry Pi
GW	LoRaWAN Gateway	SF	Spreading Factor
IN	Inner Node	WSN	Wireless Sensor Network
